# Characterization of blood-brain barrier L-arginine uptake using in situ brain perfusions in a female mouse model

**DOI:** 10.1186/s12987-026-00832-3

**Published:** 2026-06-13

**Authors:** Olivia C. Milam, Cullen P. Wolford, Geoffrey L. Pecar, Joshua J. Applegate, Maxine E. Casto, Dominic J. Gabriele, Austin S. Nestor, Paul R. Lockman

**Affiliations:** 1 Department of Basic Pharmaceutical Sciences, School of Pharmacy, West Virginia University HSC, 64 Medical Center Dr., Morgantown, WV 26505 USA; 2 School of Medicine, West Virginia University, Morgantown, WV USA

**Keywords:** L-arginine, Amino acid homeostasis, Amino acid brain transport, Blood-brain barrier, Nitric oxide, Transport kinetics

## Abstract

**Background:**

L-arginine is a critical determinant of central nervous system (CNS) function through nitric oxide (NO) production. Its uptake from plasma into brain is dependent on carrier-mediated transport across the blood-brain barrier (BBB). Transport kinetics of L-arginine BBB uptake have been assessed in rat models, but saturation constants such as maximal transport rate (*V*_*max*_), half-saturation constant (*K*_*M*_), and diffusion constant (*K*_*D*_) in mice remain unknown. The aim of this study was to determine the transporter responsible for L-arginine BBB transport and to provide a complete kinetic profile, including whole brain and regional saturation kinetics of its transport, in a female mouse model.

**Methods:**

BALBc mice were perfused with ^3^H-L-arginine using the in situ brain perfusion technique. Linear and unidirectional uptake was determined by perfusion at increasing timepoints (15-60s). Saturation kinetics were identified regionally and in whole brain by adding unlabeled L-arginine to buffer and perfusing for 45s. Sodium sensitivity was evaluated by decreasing sodium levels with replacement of cesium to maintain physiologic osmolarity. Dependence of transport on hydrogen ions was determined across ranges of pH (5.5–8) by addition of hydrochloric acid or sodium hydroxide. The transport system responsible for L-arginine BBB transport was assessed by adding inhibitors such as harmaline, N-methylmaleimide (NMM), L-homoarginine, cimetidine, and 2-amino-2-norbornanecarboxylic acid (BCH), and was further evaluated for affinity to other cationic amino acids, including L-lysine and L-ornithine. Inhibitory constants (*K*_i_) were calculated to assess the affinity of inhibitors at the transporter.

**Results:**

BBB arginine uptake showed both saturable and nonsaturable components, with a whole brain *K*_*in*_, *K*_M_ and *V*_max_ of 0.25 ± 0.02 × 10^−2^ mL/s/g, 55 ± 10 µM and 5.9 ± 0.3 nmol/min/g, respectively. Whole brain diffusion constant, *K*_*D*_, was 2.7 ± 1.0 × 10^−4^ mL/s/g. Furthermore, regional data showed cerebellar *V*_max_ was significantly higher than in cortical tissue (5.5 ± 0.6 vs. 9.3 ± 0.9 nmol/min/g). L-arginine transport was insensitive to sodium depletion and was not inhibited at pH levels 7, 7.4, or 8, but was significantly inhibited at pH 5.5. Its transport was not significantly inhibited by BCH, harmaline, NMM, or cimetidine, but was sensitive to inhibition by L-homoarginine and other cationic amino acids, including lysine and ornithine.

**Conclusion:**

The results indicate that mice predominantly use the y+ system, a cationic amino acid transporter, to transport L-arginine across the BBB. Our work supports previous characterization of BBB carrier-mediated transport of L-arginine yet extends the data by assessing complete Michaelis-Menten transport kinetics across regions and in whole brain in a female mouse model. Data further suggest species can influence BBB L-arginine transport function and there is differential need for L-arginine between brain regions. This data serves as a baseline for studies involving alterations in cationic amino acid homeostasis or altered L-arginine metabolism such as in cases of arginine auxotrophy.

**Supplementary information:**

The online version contains supplementary material available at 10.1186/s12987-026-00832-3.

## Introduction

L-arginine, a semi-essential cationic amino acid, is a critical regulator of central nervous system (CNS) function. It is predominantly metabolized by nitric oxide synthase (NOS) into nitric oxide (NO), which contribute to numerous biological processes, including the regulation of cerebral blood flow, endothelial cell function, neuronal protection, neuroinflammation, neurotransmission, and cognitive outcomes [1–6]. Nitric oxide production is tightly regulated, as its levels are implicated in pathology, including cancer, stroke, and neurodegenerative diseases [3, 7–12]. In cancer, tumor cells rewire arginine metabolism, losing ability to synthesize endogenous arginine, thereby enabling aspartate accumulation to support pyrimidine production and enhance cellular proliferation [13, 14]. This metabolic shift creates a reliance on exogenous arginine transport into cells to meet arginine demand, revealing a vulnerability currently being investigated as a therapeutic target [15, 16]. Arginine-depleting enzymes reduce plasma arginine, starving these vulnerable cancer cells of the nutrient. Glioblastoma is a tumor type with arginine auxotrophic signatures, meaning it has a heightened reliance on cellular arginine transport, raising the question of whether blood-tumor barrier transport is also altered [17–19]. In addition to potential alterations in amino acid homeostasis due to enhanced nutrient reliance, using arginine-depleting enzymes as a therapeutic may also cause differential kinetics at the barrier.

*De novo* synthesis of L-arginine occurs to a negligible degree under physiological conditions, and accordingly, supply to brain is derived from circulating plasma levels mediated by blood-brain barrier (BBB) transport. Like all amino acids, L-arginine requires carrier-mediated transport to penetrate the BBB due to its physicochemical properties, including its polarity and charge at physiological pH. L-arginine uses the y+ transport system, which selectively binds cationic amino acids such as arginine, lysine, and ornithine independent of sodium [20]. The y+ system includes transporter proteins such as the cationic amino acid transporters 1–3 (CAT1-3,SLC7A1-3). Other transporters may influence cationic amino acid uptake, including B^0,+^(SLC6A14), b^0,+^(SLC7A9, SLC3A1), y+L (SLC7A6-7) systems, and organic cation transporters (OCT, SLC22A1-3). While data identify the y+ family as the dominant system for L-arginine BBB transport, characterization of whole brain and regional saturable and nonsaturable transport constants in mice remains incomplete [20, 21]. Fidanboylu and Thomas (2024) established that BBB L-arginine transport is saturable in mice with addition of 1,000,116 nM L-arginine, but kinetic constants such as half saturation constant (*K*_M_), diffusion constant (*K*_D_), and maximal transport rate (*V*_max_) were not calculated for its transport [22]. Additionally, unidirectional transfer constant (*K*_in_) at a single time point of 10 minutes has been assessed, but *K*_in_ at earlier time points has not been calculated [23]. Therefore, establishing kinetic constants of carrier-mediated L-arginine transport into brain provides a context for investigating how alterations in this pathway may impact BBB arginine availability and transport. More specifically, identifying kinetic constants in a mouse model is necessary because many pathologies are now modeled in mice, including cancer, stroke, infection, etc. Therefore, baseline kinetics in a mouse model are necessary for comparisons for transport in pathological states such as cancer.

Herein, we hypothesized that L-arginine BBB transport in mice would be predominantly carrier-mediated and saturable through the y+ system, and that its uptake kinetics, including *K*_*M*_* and V*_max_, would differ between cortical/subcortical tissue and cerebellum. To address this, we determined transporter identity and completed a comprehensive kinetic characterization of physiological BBB L-arginine transport, including unidirectional transfer constant (*K*_in_), whole-brain and regional Michaelis-Menten transport kinetics (*V*_max_, *K*_D_, and *K*_M_), and inhibitory kinetics (*K*_i_) for transport competitors.

## Methods and materials

### Animals

All animal experiments were performed according to West Virginia University Institutional Animal Care and Use Committee protocols. Female BALB/cJ mice were purchased from Jackson Laboratory (Bar Harbor, ME). All animals were approximately 6–9 weeks and 25 g. Animals were allowed to acclimate for at least 72 hours before experimentation. Number of animals used per group are detailed in figure legends.

### In situ brain perfusion technique

#### Unidirectional and linear uptake of L-Arginine

Arginine uptake into brain was measured using the in situ brain perfusion technique, as described by Takasato et al. and modified in mice by Mittapalli et al. [24–26]. Perfusion buffer matched physiological conditions (2.4 mM NaH_2_PO_4_, 4.2 mM KCl, 24 mM NaHCO_3_, 128 mM NaCl, 1.5 mM CaCl_2_, 0.9 mM MgCl_2_, and 9 mM D-glucose) with 0.2 µCi/µL (4.7 nM) ^3^H-L-arginine (specific activity- 42.9 Ci/mmol, Revitty, Waltham, MA) and heated to 37 °C.

To determine BBB uptake, mice were anesthetized by intraperitoneal injection of ketamine (100 mg/kg) and xylazine (10 mg/kg), then perfused through the left cardiac ventricle with perfusion buffer for 15–60 seconds at a flow rate of 5 mL/minute. The right atrium was nicked to prevent recirculation of buffer. Following perfusion, mice were decapitated and brain was collected, weighed, and digested in 5 mL solvable (Revvity, Waltham, MA) in scintillation vials overnight at 55 °C. Then, 5 mL UltimaGold Liquid Scintillation Cocktail (Revvity, Waltham, MA) was added to samples. Samples were vortexed and read on a Tri-Carb Liquid Scintillation Counter (PerkinElmer, Waltham, MA). This experimental technique was followed for all experiments, with alterations to the perfusion buffer in inhibition studies. During experimentation, control animals are perfused with perfusion buffer containing 4.7 nM ^3^H-L-arginine and 92 µm ^14^C-Sucrose at physiological pH and osmolarity. Whole brain uptake of ^3^H-L-arginine was characterized using the unidirectional transfer constant, *K*_in_ (mL/s/g), and was calculated using the equation below (Eq. 1).

1 $${{\rm{Q}}_{{\rm{Br}}}}{\rm{/}}{{\rm{C}}_{{\rm{pf}}}}{\rm{ = }}{K_{{\rm{in}}}}\left( {\rm{T}} \right){\rm{ + }}{{\rm{V}}_{\rm{0}}}$$

where Q_Br_ is the measure of radioactive tracer (^3^H-L-arginine) present in brain (dpm/g), C_pf_ is the radioactivity of ^3^H-L-arginine added into physiological buffer, T is the perfusion time and V_0_ is vascular volume found at time (T) at 0 seconds. Unidirectional and linear uptake was first established by fitting data to linear regression least squares fit modeling (GraphPad Software, La Jolla, California) where Q_br_/C_pf_ is measured in response to time (15, 30, 45, 60 seconds). To determine a single *K*_in_ from experimental data, Eq. 1 was rearranged to mathematically solve for *K*_in_ at *T* = 45s.

#### Saturable and nonsaturable parameters of L-Arginine transport

Saturable and nonsaturable parameters of ^3^H-L-arginine transport were determined in whole brain using the in situ brain perfusion technique with unlabeled L-arginine added to perfusion buffer with final unlabeled concentrations of 0.1, 10, 25, 35, 50, 100, 150, 250, 300, 450 µM.

In addition to whole-brain analysis, saturable and nonsaturable components by region were determined. After 45s perfusion, brains were collected and separated into cortical, subcortical, and cerebellum tissue on a PBS-wetted Whatman paper on ice. The regions were then weighed and prepped for liquid scintillation counting as mentioned above. Subcortical, cortical, and cerebellum mean ± SD weights were 0.06 ± 0.01, 0.3 ± 0.03, and 0.05 ± 0.01, respectively.

Michaelis-Menten transport kinetics were calculated, where the half-saturation constant of L-arginine at BBB is *K*_M_ (µM), diffusion constant, *K*_D_ (mL/s/g), is L-arginine movement in the absence of a carrier-mediated transporter, and *V*_max_ is the maximum transport rate for ^3^H-L-arginine (nmol/min/g) at the transporter. Diffusion constant was determined by fitting data to a least-squares fit non-linear regression model to Eq. 2. Michaelis-Menten kinetic constants were determined by fitting data to a nonlinear regression of the Michaelis-Menten curve and using the following equations (Eqs. 2 and 3)

2 $${K_{{\rm{in}}}} = \left[ {{V_{{\rm{max}}}}/\left( {{K_{\rm{M}}} + {{\rm{C}}_{{\rm{pf}}}}} \right)} \right]{\rm{ }} + {K_{\rm{D}}}$$

3 $${J_{{\rm{in}}}} = \left( {{V_{{\rm{max}}}}\left( {{{\rm{C}}_{{\rm{pf}}}}} \right)} \right)/\left( {{K_{\rm{M}}} + {\rm{ }}{{\rm{C}}_{{\rm{pf}}}}} \right) + {K_{\rm{D}}}\left( {{{\rm{C}}_{{\rm{pf}}}}} \right)$$

Arginine influx (*J*_in_, nmol/min/g) was determined by multiplying unidirectional uptake constant (*K*_in_) by labeled plus unlabeled concentration of L-arginine.

#### L-arginine uptake as modeled by Lineweaver burk plot

Saturation data obtained were transformed to calculate *K*_M_ and *V*_max_ by taking the reciprocals of the concentration of unlabeled L-arginine and the total influx, and the resulting data were plotted to fit a Lineweaver-Burk plot. Michaelis-Menten values were extrapolated using a simple linear regression line and the equation below, where *K*_M_ is the negative reciprocal of the x-intercept and *V*_max_ is the reciprocal of the y-intercept (Eq. 4).

4 $$1/{{\rm{J}}_{in}} = {K_{\rm{M}}}/{V_{{\rm{max}}}}\cdot{\left(1/ {\rm{C}} \right)\,} + \,1/{V_{{\rm{max}}}}$$

#### Inhibitory kinetics of L-arginine transport

Transport inhibition kinetics were determined using the in situ brain perfusion at 45s in a series of separate experiments, each assessing sensitivity to manipulations to perfusion buffer, including altering sodium concentration, adjusting pH, or adding transport inhibitors. 3N hydrochloric acid was titrated to bring perfusion buffer pH to 5.5–7. 1N sodium hydroxide was added to reach a pH of 8. Sodium was reduced (154.4 mM vs. 26.4 mM) by replacement of sodium chloride with cesium chloride to maintain osmolarity within physiological range [27]. To measure BBB integrity, 0.05 µCi/µL (92 µM) ^14^C-Sucrose (specific activity- 543 mCi/mmol, Moravek, Brea, California) was added to perfusion buffer for all experimental groups and control perfusions. Unlabeled L-lysine (70 µM), and L-ornithine (110 µM) were added during preparation of buffer based on previously published *K*_M_ values at the rat BBB [20]. To further determine the identity of the predominant transporter, 2-amino-2-norbornanecarboxylic acid (BCH) (2 mM), harmaline (100 µM), N-methylmaleimide (NMM) (100 µM), and L-homoarginine (50 µM) were added, which are inhibitors to B0,+, b0,+, y+L, and y+ systems, respectively [21, 28–32]. Additionally, cimetidine (25 µM), an inhibitor to OCT 1 and 2, was included [33]. *K*_i_ (inhibitory constants) were determined using Eq. 5.

5 $$\left( {\left( {{K_{{\rm{in0}}}} - {K_{\rm{D}}}} \right)} \right)/\left( {\left( {{K_{{\rm{ini}}}} - {K_{\rm{D}}}} \right)} \right) = 1 + {{\rm{C}}_{\rm{i}}}/{K_{\rm{i}}}$$

where *K*_in0_ is ^3^H-L-arginine *K*_in_ in the absence of an inhibitor, *K*_ini_ is the *K*_in_ in the presence of an inhibitor, C_i_ is the concentration of inhibitor added, and *K*_i_ is defined as the inhibitor concentration that decreases ^3^H-L-arginine saturable influx by 50%.

### Statistical analyses

Values were expressed as mean ± SEM for n separate determinants. Data were evaluated using GraphPad Prism software program version 10.6.1 (GraphPad Software, La Jolla, California). Unpaired T-tests with Welch’s correction or Brown-Forsythe and Welch ANOVA tests were employed for statistical analysis. Goodness-of-fit of the experimental models was assessed using an F-test on the residual mean squares. Robust regression and outlier tests (ROUT) were performed (Q = 1%), but no outliers were removed. Normality of data was analyzed using the Shapiro-Wilk test. Statistical significance was assessed using a *p*-value < 0.05.

## Results

### L-arginine transport at the BBB is linear and unidirectional for up to 60s

**Fig. 1:**
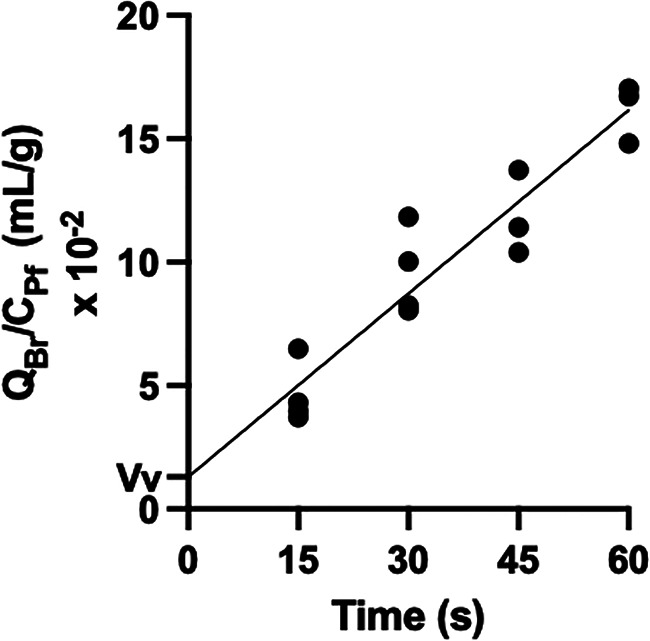
Time course of ^3^H-L-arginine uptake into whole brain during perfusion with physiological buffer (Na = 150 mM). Perfusions were performed at 15-60s time points with 0.2 µCi/µL ^3^H-L-arginine (4.7 nM). Line represents linear regression modeled to Eq. 1 with least squares fit. Each point represents one mouse. (R^2^ = 0.9, *p* < 0.0001)

To determine linear and unidirectional transport of L-arginine at the BBB, a 60s-time course of ^3^H-L-arginine uptake was performed, and the *K*_in_ of 0.25 ± 0.02 × 10^−2^ mL/s/g (Eq. 1) was apparently linear (Fig. 1). The vascular volume (V_0_) extrapolated from best fit linear regression was 1.3 ± 0.9 × 10^−2^ mL/g, indicating BBB integrity was intact during the experiments and was consistent with previously reported vascular volumes in mice [34].

### L-arginine transport has saturable and nonsaturable parameters

**Fig. 2:**
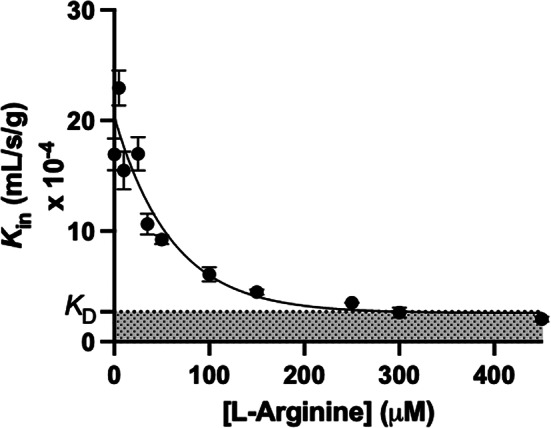
Unidirectional uptake constants (*K*_in_) of ^3^H-L-arginine in whole brain. Dependence of increasing unlabeled L-arginine concentration (0.0047–450 µM) in physiological buffer. Curve represents best fit nonlinear regression to Eq. 2 where *K*_D_ = 2.7 ± 0.9 × 10^−4^ mL/s/g. Each point is mean ± SEM for *n* = 3–4 mice

To elucidate the concentration dependence of L-arginine transport, mice were perfused with a physiological buffer containing 0.0047 µM-450 µM unlabeled L-arginine. As shown in Fig. 2, L-arginine uptake decreased by 86% at concentrations above 200 µM, consistent with a saturated transport system. Fitting the data to a non-linear regression model, *K*_D_ is 2.7 ± 0.9 × 10^−4^ mL/s/g (Fig. 2). This data indicates approximately 90% of L-arginine brain penetration is carrier-dependent, with the remainder moving by diffusion.

**Fig. 3:**
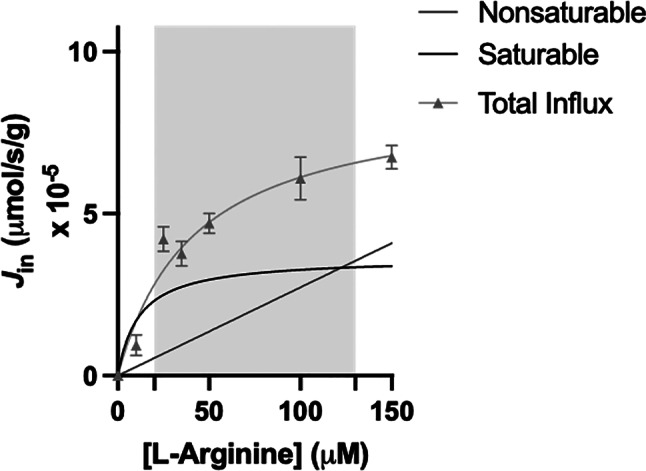
Curves represent brain total influx, saturable influx, and nonsaturable influx as a function of unlabeled L-arginine concentration (0.0047–150 µM) modeled by Eq. 3. each point is mean ± SEM for *n* = 3–4 mice. Highlighted is the range of human plasma arginine concentration (21–137 µM) [35–39]

The data for BBB ^3^H-L-arginine uptake best fit a model containing both a saturable (Michaelis-Menten) and a nonsaturable component (transport in the absence of carrier). Kinetic values were obtained based on the model fit to Eq. 3 and plotted in Fig. 3. From 0 to 130 µM accumulation in the brain is through a saturable transporter, at concentrations above 130 µM transporters are fully occupied and free diffusion is measurable. Using a two-component model, *V*_max_ and *K*_M_ were determined to be 5.9 ± 0.3 nmol/min/g and 55 ± 10 µM, respectively.

Regional assessment of L-arginine BBB uptake indicated saturable transport (Table 1) and significant variation in *V*_max_ in cortical, subcortical, and cerebellar regions, ranging from 5.5–9.3 nmol/min/g (Table 2). Cerebellar *V*_max_ was significantly higher than in cortical tissue (*p* < 0.05), with an increase of 70%. *K*_M_ was unchanged across groups, with an overall average of 58 ± 21 µM.

**Table 1:** Whole brain and regional *K*_in_ values × 10^4^ at 45s in response to increasing unlabeled L-arginine concentrations. Data represent mean ± SEM for 3–4 mice

Unidirectional and linear uptake of ^3^H-L-arginine in response to increasing concentrations of unlabled L-arginine								
	Perfusate arginine concentration (µM)							
Region	0.0047	25	35	50	100	150	350	450
Whole Brain	25 ± 0.2	17 ± 1.0	13 ± 1.9	9 ± 0.3	6 ± 0.6	4 ± 0.2	3 ± 0.4	2 ± 0.3
Cortical	17 ± 2.0	13 ± 1.0	13 ± 0.2	9 ± 1.8	6 ± 1.5	5 ± 0.3	3 ± 0.3	2 ± 0.3
Subcortical	19 ± 2.0	16 ± 0.6	14 ± 0.8	10 ± 0.9	8 ± 1.0	6 ± 0.4	3 ± 0.4	2 ± 0.4
Cerebellum	20 ± 3.0	17 ± 1.0	12 ± 1.7	15 ± 2.6	7 ± 0.7	8 ± 0.3	4 ± 0.6	3 ± 0.1

**Table 2:** Regional *V*_max_, *K*_D_, and *K*_M_ for ^3^H-L-arginine BBB L-arginine transport. Values are mean ± SEM for 3–4 animals. Statistical analysis includes an extra-sum-of-squares F-test for best-fit parameters, * indicates *p* < 0.05 from cortical tissue

Regional Pharmacokinetic Parameters for BBB ^3^H-L-arginine transport			
Region	V_max_ (nmol/min/g)	K_D_ (mL/s/g)	K_M_ (µM)
Cortical	5.5 ± 0.6	2.1 ± 0.4	45 ± 19
Subcortical	6.9 ± 0.7	2.5 ± 0.7	48 ± 17
Cerebellum	9.3 ± 0.9*	3.2 ± 0.5	78 ± 24

### L-arginine transport kinetics fitted to the Lineweaver-Burk plot

**Fig. 4:**
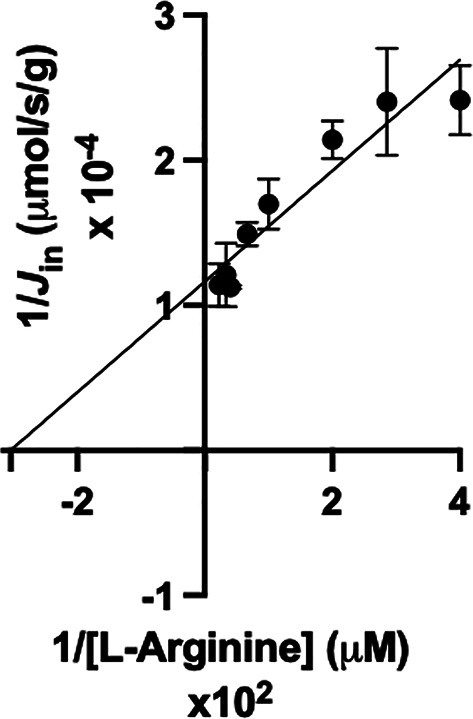
Sensitivity of BBB ^3^H-L-arginine transport to increasing unlabeled L-arginine concentrations as represented by a linear Lineweaver-Burk plot. Each point represents mean ± SEM for 3–4 animals

L-arginine BBB transport data can also be fit to a Lineweaver-Burk plot, which describes Michaelis-Menten kinetics, using linear analysis. As shown in Fig. 4, Michaelis-Menten parameters are extrapolated from the plot using Eq. 4. This secondary analysis shows *V*_max_ and *K*_M_ were 5.2 ± 0.5 nmol/min/g and 32 ± 8 µM, respectively. These data are similar to the values obtained using the nonlinear regression Michaelis-Menten curve.

### L-arginine transport occurs independently of sodium and at pH > 5.5

**Fig. 5:**
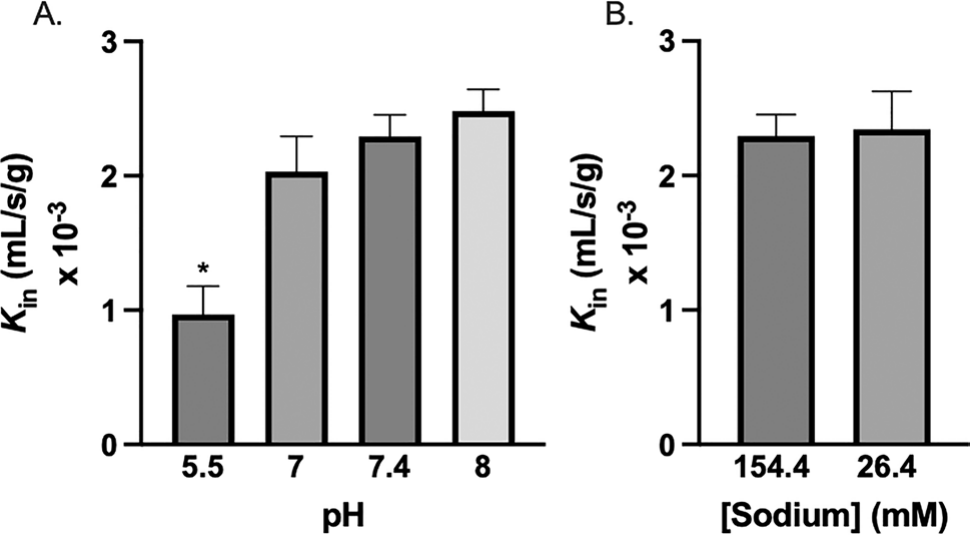
**A**. ^3^H-L-arginine transport into brain at varying pH levels (5.5–8) **B**. Transport in the presence of reduced sodium (154.4 mM v. 26.4 mM). Data represent mean ± SEM for *n* = 5 mice. * represents statistical significance from pH 7.4 (*p* < 0.05)

The pH and sodium dependence of ^3^H-L-arginine BBB transport activity was determined. A decrease in pH to 5.5 significantly inhibited L-arginine uptake by 54% compared to physiological pH (7.4) (*K*_in_ = 1.0 ± 0.2 × 10^−3^ mL/s/g) (Fig. 5). L-arginine uptake was sodium-independent, with *K*_in_ values of 2.3 ± 0.2 × 10^−3^ mL/s/g vs. 2.3 ± 0.3 × 10^−3^ mL/s/g. No significant effects (*p* > 0.05) occurred when sodium chloride was replaced with cesium chloride. ^14^C-Sucrose values remained within physiologic vascular volume range (1.0–1.7 × 10^−2^ mL/g), indicating intact BBB (Supplementary Data 1).

### L-arginine uptake is inhibited by L-lysine, L-ornithine, and L-arginine

**Fig. 6:**
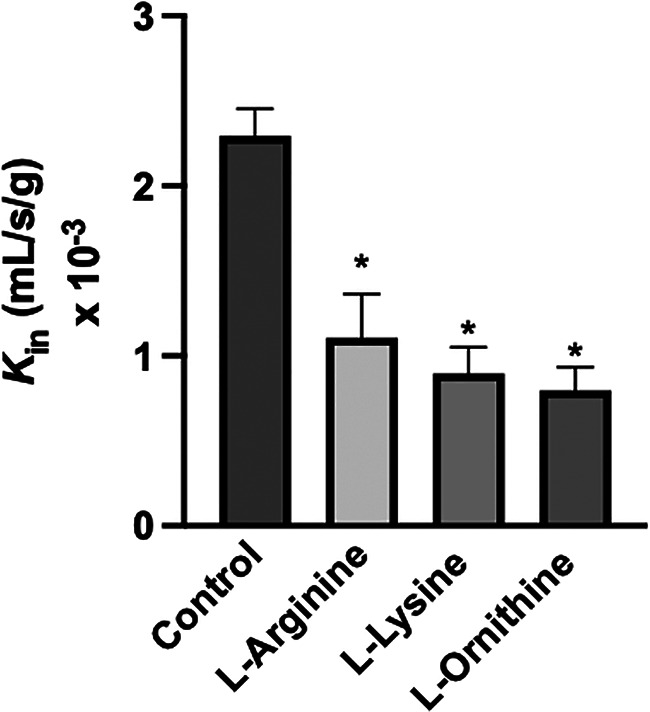
^3^H-L-arginine transport in the presence of L-lysine (70 µM), L-ornithine (110 µM), and L-arginine (31 µM). Data represent mean ± SEM for *n* = 5 mice. * indicate significance (*p* < 0.05) compared to control

Co-perfusions with physiological buffers containing amino acids dependent on the y+ system were performed to elucidate inhibitory kinetics. Addition of L-lysine (70 µM) and L-ornithine (110 µM) at their previously published *K*_M_ values significantly (*p* < 0.05) reduced L-arginine uptake by 60% (*K*_in_ = 0.9 ± 0.2 × 10^−3^ mL/s/g) and 57% (*K*_in_ = 0.8 ± 0.1 × 10^−3^ mL/s/g) respectively (Fig. 6). Likewise, L-arginine (31 µM) decreased transport by 52% (*K*_in_ = 1.1 ± 0.3 × 10^−3^) Inhibitory constants were 33 ± 13 µM for arginine, 37 ± 15 µM for lysine, and 44 ± 15 for ornithine (Eq. 5). Barrier integrity remained intact with vascular volumes between 1.1–1.4 × 10^−2^ mL/g (Supplementary material 2).

### Transport of L-arginine is dependent on the y+ system

**Fig. 7:**
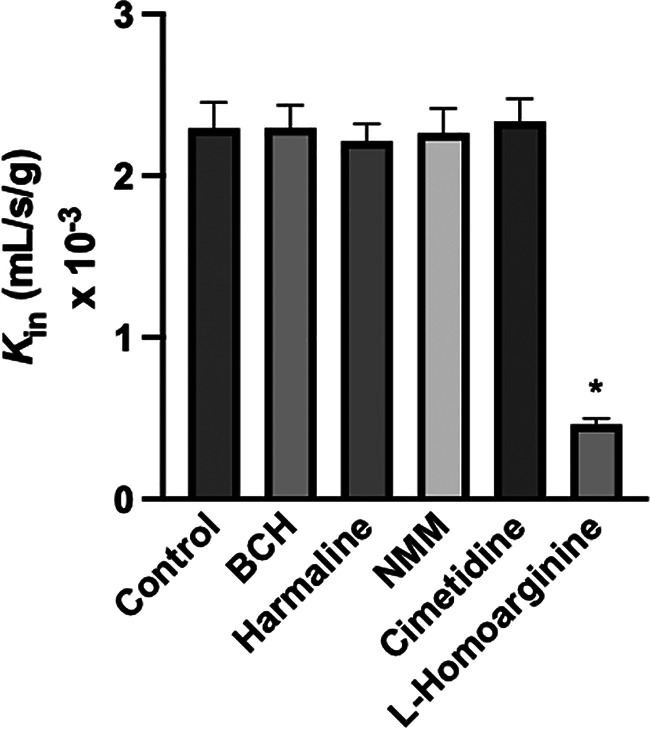
^3^H-L-arginine unidirectional uptake (K_in_) treated with 2 mM BCH (B^0,+^ inhibitor), 100 µM harmaline (b^0,+^ inhibitor), 100 µM N-methylmaleimide (y+L inhibitor), 25 µM cimetidine (Oct 1 and 2 inhibitor) and 50 µM L-homoarginine (y+ inhibitor). Data represent mean ± SEM for *n* = 5–7 per group. * represents statistical significance (*p* < 0.05)

To confirm the transporter responsible for BBB L-arginine uptake, known inhibitors were added to the perfusion buffers (Fig. 7). BCH, a known inhibitor to B^0,+^, had no significant effect on ^3^H-L-arginine uptake (*K*_in_ = 2.3 ± 0.2 × 10^−3^ vs. 2.3 ± 0.1 × 10^−3^ mL/s/g). Likewise, harmaline (*K*_in_ = 2.2 ± 0.1 × 10^−3^), NMM (*K*_in_ = 2.3 ± 0.2 × 10^−3^), and cimetidine (*K*_in_ = 2.3 ± 0.1 × 10^−3^), known inhibitors to b^0,+^, y+L, and OCT respectively, did not alter transport (*p* > 0.05). Addition of L-homoarginine, a known substrate for the y+ system, significantly inhibited BBB L-arginine transport (*p* < 0.05) by 78% (*K*_in_ = 0.5 ± 0.04 × 10^−3^). The concentration of L-homoarginine required to reduce transport by 50% is 5.3 ± 1.1 µM. Vascular volume remained within physiological levels, indicating an intact BBB (Supplementary material 2).

## Discussion

We demonstrate BBB transport of L-arginine has saturable and nonsaturable parameters that are independent of sodium and significantly inhibited at pH levels below 5.5. These results are consistent with previous characterization of BBB L-arginine transport in other mammals [20, 21, 40]. Additionally, we demonstrate L-arginine transport has an affinity for other cationic amino acids such as L-lysine and L-ornithine. Herein, the novelty of this study is threefold. First, to the best of our knowledge, we are among the first to study L-arginine BBB uptake in a female mouse model using linear regression analysis to assess its initial rate of transport under unidirectional and linear uptake. Second, we performed a Lineweaver-Burk analysis of L-arginine BBB transport and third, we identify whole-brain and regional Michaelis-Menten kinetics in a mouse model, which has not been previously established.

We aimed to characterize the L-arginine transporter by assessing its sensitivity to buffer modifications (manipulation of pH and sodium) and to competitors (addition of inhibitors to the perfusion buffer). Consistent with other data, the transport mechanism of L-arginine at the BBB is sodium-independent and functions at pH > 5.5 (Fig. 5). We did not observe differences in L-arginine uptake (Fig. 6) when sodium was reduced and replaced with osmolar equivalents. Additionally, *c*hanges in pH did not significantly affect *K*_in_ except at pH values below 5.5, consistent with Oldendorf and colleagues [41]. ^14^C-Sucrose was used to monitor vascular integrity, ensuring that changes in transport were not secondary to BBB disruption. (Supplementary material 1) Transporters have characteristics that guide their activity, pH and sodium dependence being two descriptors. By assessing sensitivity to alterations in these parameters, we can begin to identify transporters not involved in L-arginine uptake, such as sodium-dependent transport systems. Moreover, transport can be influenced by hydrogen ions if it relies on their exchange. For example, multidrug and toxic compound extrusion (MATE) transporters are proton-coupled antiporters whose function is driven by proton movement across the membrane [42]. The y+ system is not a known hydrogen exchanger and is not dependent on a hydrogen gradient. Therefore, inhibition of L-arginine transport at pH 5.5 can be explained by alternative mechanisms. First, excess protons could interact with residues of the binding pocket. The first cryo-EM structure of mammalian CAT-1 revealed that serine 120 and aspartic acid 263 were key residues initiating substrate binding, particularly aspartic acid 263, which served as the negative anchor for positively charged substrates [43]. However, when ionizable residues are trapped within hydrophobic pockets, there is a shift in pKa, with acidic amino acids typically showing increased pKa [44]. It is probable that at pH 5.5, the carboxylic acid of aspartic acid 263 becomes protonated and loses its overall negative charge, disabling substrate anchoring, thus decreasing L-arginine transport. Alternatively, L-arginine transport is membrane potential-dependent, meaning fluctuations in pH could cause depolarization and, accordingly, decrease transport [21]. The decrease of L-arginine transport at pH 5.5 is consistent with that of the y+ system and has implications in cancer models due to acidic tumor microenvironments.

Next, cationic amino acids, L-lysine and L-ornithine, significantly inhibit L-arginine BBB uptake. Ranked apparent affinity for the transporter is arginine > lysine >ornithine, with inhibitory constants of 33 ± 13 µM, 37 ± 15 µM, and 44 ± 15 µM. These data indicate a high-affinity transporter for all three cationic amino acids. In humans, plasma concentrations of the cationic amino acids are 21–137 µM, 150–250 µM, and 19–81 µM for arginine, lysine, and ornithine, respectively [35, 45, 46]. At physiological concentrations, although arginine has the highest affinity, lysine may outcompete it due to its higher baseline plasma concentration.

Moreover, to determine if the y+ system is the predominant BBB transporter of L-arginine, inhibitors of other amino acid systems known to interact with cationic amino acids were added to perfusion buffer. In addition to the y+ family, cationic amino acids can interact with y+L, B^0,+^, and b^0,+^ transporters [40, 47, 48]. The y+L system is a high-affinity, large neutral amino acid transporter with overlapping specificity for cationic amino acid transport. This interaction is dependent upon ionic composition of buffer/media where affinity in the presence of Na+ is greater for neutral amino acids such as leucine and isoleucine compared to cationic amino acids. Both B^0,+^, and b^0,+^ are classified as broad substrate carriers and like y+L, they have an affinity for both cationic amino acids and neutral amino acids; however, these systems preferentially transport branched neutral amino acids. The y+, y+L, and b^0,+^ systems are sodium-independent, while B^0,+^ is sodium-dependent. Thus, insignificant changes (*p* > 0.05) in *K*_in_ with reduced sodium confirm that B^0,+^ is not a dominant transporter of L-arginine at the BBB. Further, co-perfusion with BCH, a known inhibitor of B^0,+,^ did not significantly affect L-arginine transport, providing additional confirmation. Addition of harmaline (b^0,+^ inhibitor), NMM (y+L inhibitor), and cimetidine (OCT 1 and 2 inhibitor) did not alter brain L-arginine uptake (Fig. 7), suggesting that the y+L, b^0,+^, and OCT systems are also not responsible for the majority of brain L-arginine uptake. In contrast, addition of L-homoarginine, a known inhibitor of the CAT family, significantly reduced L-arginine transport [30]. Therefore, because L-arginine transport is sodium independent, sensitive to low pH, reduced by competition with unlabeled homoarginine, arginine, lysine, and ornithine, and is unaffected by BCH, harmaline, NMM, or cimetidine, it is suggested that the predominant L-arginine transporter at the female mouse BBB is the cationic amino acid transporter family.

Further, our study examined unidirectional and linear uptake of ^3^H-L-arginine using the modified in situ brain perfusion technique. For calculations in this study, *K*_in_ was used instead of permeability surface area product (PA). While *K*_in_ and PS measure rates of solute transfer to brain tissue, the distinction between the two constants is perfusion flow. To penetrate the BBB, solutes must have favorable physicochemical properties, including a balance between lipophilicity and hydrophilicity. Small lipophilic solutes freely diffuse across the membrane, where flow rates influence uptake, and vascular surface area need to be considered [49]. The limitation for L-arginine BBB penetrance is not flow-limited, but it is extraction-limited (*K*_in_ is less than 20% of apparent blood flow) at the membrane due to its water-soluble properties.

A recent study has determined *K*_in_ of L-arginine using an in situ brain perfusion technique in a male mouse model [23]. Our data complements the earlier report by providing insight into uptake at earlier time points not captured in their data. Variations in *K*_in_ are in part due to differences in composition of perfusion buffer and the utilized mathematical method. First, the previously published experiments included 1 g/L of serum albumin in perfusion, resulting in an apparently lower K_in_ due to protein binding [50, 51]. Next, our data uses a different method to calculate K_in._ We capture data during short perfusion periods (15-60s) to measure linear and initial uptake across the BBB, whereas previous research captures it at a single point of 10 minutes. Second, the mathematical model in our data corrects for *V*_*i*_ by extrapolating the y-intercept (Eq. 1). Further, we observed the volume of distribution of L-arginine in brain was approximately 30-fold higher than that of sucrose (our linear analysis extrapolated to 2.5 minutes), which is within a factor of two compared to the other data. Ultimately, these methodological differences together provide complementary insights into L-arginine BBB transport. While methodological differences account for variance in *K*_in_, this does not rule out the potential for sex-dependent regulation of L-arginine transport through CAT-1 at the BBB, warranting a head-to-head investigation in males and females.

Next, to determine saturation kinetics, increasing levels of unlabeled L-arginine were added to the perfusion buffer. Our data suggest the bulk of L-arginine BBB transport is carrier-mediated, with only 10% mediated by diffusion (Fig. 2). A saturable component of L-arginine uptake has been reported in in vitro studies using various cell types, including in retinal pericytes, glial cells, brain astrocytes, and brain endothelial cells [52–55]. Further, kinetic constants of saturable L-arginine transport have been identified in vivo, but only in rat models [20, 41, 56, 57]. Using the in situ brain perfusion technique in a rat model, *K*_M_ and *V*_max_ were 56 ± 9 µM and 23 ± 3 nmol/min/g, respectively [20]. Our *K*_M_ values are within the margin of previously published data sets, but our *V*_max_ is approximately 4-fold lower. L-arginine is a substrate for many enzymes, and accordingly, differential enzyme activity between species can cause variation in maximal transport rates and BBB transporter expression. It has been reported rats have higher NOS activity than mice, which could explain the higher *V*_max_ [58]. More efficient transport and increased CAT expression provide more L-arginine substrate. The binding pocket of the y+ system (specifically CAT-1) is highly conserved across species, with >90% similarity; therefore, if the same isoform is expressed at the BBB, changes in *K*_M_ are not expected [59–61].

Regional saturation data indicate no difference between half-saturation constants amongst the regions. Consistent *K*_M_ values indicate the transporter’s affinity for arginine is the same regardless of brain area, suggesting the same transporter isoform is seen throughout the brain. In contrast, there was a significant difference (*p* < 0.05) in *V*_max_ between cerebellar and cortical regions (5.5 ± 0.6 vs. 9.3 ± 1.0 nmol/min/g). Similar findings were observed when comparing L-arginine uptake in cerebellar synaptosomes versus cortical synaptosomes [62]. This likely reflects a specialized adaptation to regional metabolic demand as the cerebellum possesses high levels of NOS activity. An elevated *V*_max_ indicates a higher transporter density is needed to maintain precursor pools for robust nitrergic signaling [58, 62]. In addition, cerebellar tissue has higher levels of tetrahydrobiopterin, an essential cofactor for NOS activity, further supporting the increased need for arginine [63]. Alternatively, differences in vasculature density may be a contributor to *V*_max_ differences. Data exist which evaluate numerical and length densities of vessels in different regions of the brain. The data show both microvessel density and length density of cerebellum gray matter (296 mm^−2^) is slightly higher than cerebral cortex (255 mm^−2^) [64]. A higher surface area creates a larger interface for metabolic exchange and potentially increased transporter expression. Furthermore, diffusional components did not differ significantly between cortical and cerebellar regions. However, of total uptake for each region, only ~12% and ~16% of transport was nonsaturable. To our knowledge, we are the first to determine regional saturation kinetics, including *V*_max_, *K*_*M*_, *and K*_*D*,_ in a murine model using the in situ brain perfusion technique, highlighting differential uptake that may reflect metabolic need.

Furthermore, comparison of whole brain total, saturable, and nonsaturable influx showed that below 130 µM, the predominant mechanism of transport is saturable. It is believed that at levels above this threshold, the system is nearly fully occupied, and movement relies on both transport and diffusion. Physiological plasma concentrations of L-arginine are ~140 µM in mice, and human concentrations range from 21 to 137 µM [35–39]. Our influx data suggest that at physiological concentrations, L-arginine transporters function between 50 and 75% of maximal transport capacity (highlighted in Fig. 3).

Enzyme kinetics can be modeled in multiple ways, including the hyperbola curve, Lineweaver-Burk plot, and the Eadie-Hofstee plot. We transformed whole brain kinetic data at the BBB to fit the Lineweaver-Burk plot (Fig. 4). This, in large part, agrees with values extrapolated by the nonlinear regression curve (*V*_max_ = 5.2 ± 0.5 nmol/min/g, *K*_M_ = 32 ± 8 µM). An advantage of this plot is its ease of data visualization. It provides a simple method for determining BBB transport kinetics, such as *K*_M_ and *V*_max,_ via slope and intercepts, and for differentiating inhibition types. Establishing baseline kinetic parameters of L-arginine BBB transport is critical, as constants may be altered in disease states.

Amino acid homeostasis is normally tightly regulated, which can be disrupted in numerous pathologies. For example, in metabolic disease, such as hyperargininemia caused by arginase I deficiency, plasma arginine levels reach 300 µM or higher [65]. Patients with hyperargininemia display neurological deficits, including learning and development delay, seizures, and diplegia [66]. The primary mechanism is proposed to be ammonia accumulation; however, neurological symptoms occur even in its absence, suggesting a potential secondary mechanism. Excess plasma arginine may be responsible, as elevated levels can be toxic and can alternatively reduce lysine and ornithine bioavailability to the brain by increasing competition for transporters. Furthermore, these patients exhibit ataxia, a symptom associated with cerebellar damage [67]. Our regional data suggest the cerebellum may be more sensitive to alterations in arginine metabolism, particularly if increased arginine is available, there could be excessive levels of NO. Additionally, metabolic disease may not be the only pathology associated with differential accumulation of cationic amino acids.

Arginine metabolism is also relevant in arginine auxotrophic cancer, as previously mentioned, which is currently being targeted by investigational arginine-depleting enzymes, such as arginine deaminase (ADI-PEG20) and arginase I [17]. These drugs metabolize plasma arginine into citrulline and ornithine, limiting arginine availability and starving tumors of the nutrient. These enzymes are currently in clinical trials for treating glioblastoma, in which extraction of arginine from the blood is critical for meeting metabolic demands [68]. The drug depletes plasma arginine pools, thus decreasing the amount that crosses the BBB and inducing mitochondrial dysfunction in cancer cells [13, 17]. Treatment with ADI-PEG20 decreases plasma arginine to levels below 10 µM [68, 69]. Based on our data, *J*_in_ at 10 µM L-arginine has a transport rate of 0.6 ± 0.2 nmol/min/g (Fig. 3), suggesting that after treatment, transport will be functioning at 1/10 of max capacity. Data also suggests a decrease in arginine from ADI-PEG20 can cause an increase in lysine and ornithine in the brain. Collectively, these findings underscore the importance of investigating BBB amino acid transport and homeostasis, particularly in disease states characterized by altered arginine metabolism. By defining the regionalized and saturable nature of arginine transport, this study provides kinetic framework necessary to understand the BBB not as a static wall, but as a dynamic and adaptive interface tuned to the specific metabolic demands of the CNS. This dataset sets a foundation for comparing uptake in models of altered arginine metabolism.

This study has limitations. L-arginine BBB transport was analyzed in female mice only, as previous literature has been published in male models. However, a direct comparison between this data and earlier data is limited by differences in experimental design. Because transporter expression is hormone-sensitive, a direct comparison of L-arginine uptake within the same study is warranted [70]. Further, the in situ brain perfusion techniques is commonly used for determining the mechanism of drug and nutrient uptake across the BBB, but it is limited. For example, the technique bypasses systemic circulation and accordingly, there is no influence of peripheral organs, hormones, or other amino acids on the transport kinetics captured. Lastly, we identify that the transport family is y+, but did not further address what specific carrier is used (CAT1-3). Further analysis on the transport isoform is warranted.

## Conclusion

In summary, our study demonstrates L-arginine transport kinetics are influenced by brain region, with the cerebellum having a higher *V*_max_. Data further suggest that species can directly alter kinetics, a finding important when assessing transport-associated work preclinically. While our work examined L-arginine uptake in normal brain, investigation of its transport in CNS pathologies such as arginine auxotrophic cancer is warranted [71].

## Electronic supplementary material

Below is the link to the electronic supplementary material.

Supplementary Material 1

## Data Availability

The original contributions presented in this study are included in the article and supplementary data; further inquiries can be directed to the corresponding author, Dr. Paul Lockman.

## Abbreviations

BBB: Blood-Brain Barrier
CNS: Central Nervous System
NO: Nitric Oxide
NOS: Nitric Oxide Synthase
*K*_in_: Unidirectional Transfer Constant
*K*_D_: Diffusion Constant
*V*_max_: Maximum Transport Rate
*K*_M_: Half Saturation Constant
*J*_in_: Influx
*K*_i_: Inhibition Constant
