# Correction: Characterization of blood-brain barrier L-arginine uptake using in situ brain perfusions in a female mouse model

**DOI:** 10.1186/s12987-026-00839-w

**Published:** 2026-07-19

**Authors:** Olivia C. Milam, Cullen P. Wolford, Geoffrey L. Pecar, Joshua J. Applegate, Maxine E. Casto, Dominic J. Gabriele, Austin S. Nestor, Paul R. Lockman

**Affiliations:** 1 Department of Basic Pharmaceutical Sciences, School of Pharmacy, West Virginia University HSC, 64 Medical Center Dr., Morgantown, WV 26505 USA; 2 School of Medicine, West Virginia University, Morgantown, WV USA

**Correction: Fluids Barriers CNS 23, 81 (2026)**

10.1186/s12987-026-00832-3

Following publication of this article, the authors identified an error in Equation 4.

The equation was published as:

$${1 \mathord{\left/{\vphantom {1 {{{\mathrm{V}}_{\mathrm{0}}}}}} \right.\kern-\nulldelimiterspace} {{{\mathrm{V}}_{\mathrm{0}}}}}\, = \,{{{{\mathrm{K}}_{\mathrm{M}}}} \mathord{\left/{\vphantom {{{{\mathrm{K}}_{\mathrm{M}}}} {{{\mathrm{V}}_{{\mathrm{max}}}}}}} \right.\kern-\nulldelimiterspace} {{{\mathrm{V}}_{{\mathrm{max}}}}}}\left[ {\mathrm{C}} \right]\,{\text{ + }}\,{{\mathrm{1}} \mathord{\left/{\vphantom {{\mathrm{1}} {{{\mathrm{V}}_{{\mathrm{max}}}}}}} \right.\kern-\nulldelimiterspace} {{{\mathrm{V}}_{{\mathrm{max}}}}}}$$

The correct equation is:

$${1 \mathord{\left/{\vphantom {1 {{\mathrm{Jin}}}}} \right.\kern-\nulldelimiterspace} {{\mathrm{J}}}}_{in}\, = \,{{{{\mathrm{K}}_{\mathrm{m}}}} \mathord{\left/{\vphantom {{{{\mathrm{K}}_{\mathrm{m}}}} {{{\mathrm{V}}_{{\mathrm{max}}}}\,}}} \right.\kern-\nulldelimiterspace} {{{\mathrm{V}}_{{\mathrm{max}}}}\,}} \cdot \,\left( {{1 \mathord{\left/{\vphantom {1 {\mathrm{C}}}} \right.\kern-\nulldelimiterspace} {\mathrm{C}}}} \right)\,{\text{ + }}\,{{\mathrm{1}} \mathord{\left/{\vphantom {{\mathrm{1}} {{{\mathrm{V}}_{{\mathrm{max}}}}}}} \right.\kern-\nulldelimiterspace} {{{\mathrm{V}}_{{\mathrm{max}}}}}}$$

All analyses, calculations, and results presented in the manuscript remain correct.

The original article has been updated.

