# Supplementary material for: Characterization of blood-brain barrier L-arginine uptake using in situ brain perfusions in a female mouse model
Source: Fluids Barriers CNS. 2026 Jun 13;23:81. doi: 10.1186/s12987-026-00832-3 (PMC13270607; doi:10.1186/s12987-026-00832-3)
Supplement: Supplementary file 1 — Supplementary Material 1 [file 12987_2026_832_MOESM1_ESM.pptx]

## Slide 1
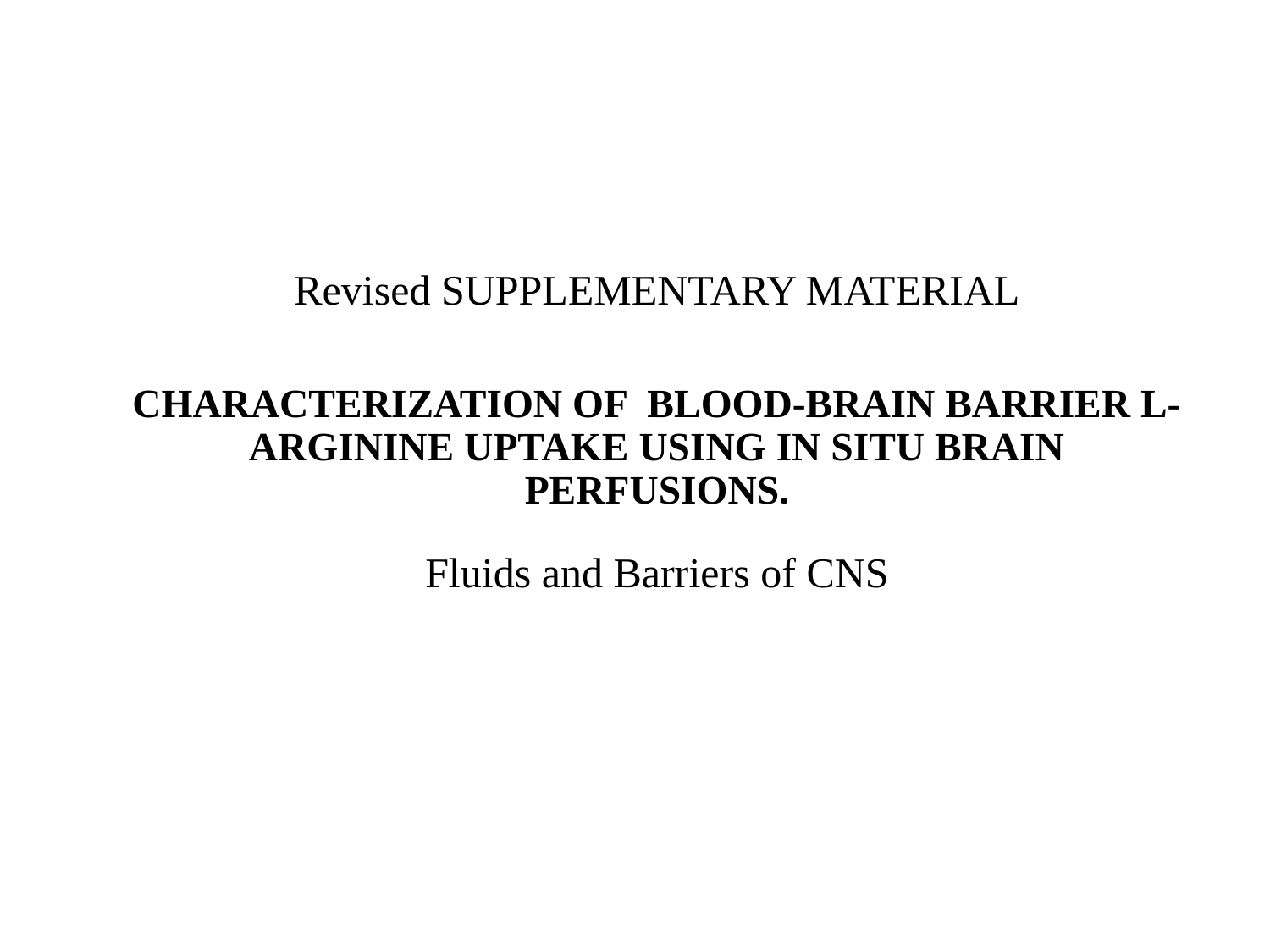

Revised SUPPLEMENTARY MATERIAL
CHARACTERIZATION OF BLOOD-BRAIN BARRIER L-ARGININE UPTAKE USING IN SITU BRAIN PERFUSIONS.
Fluids and Barriers of CNS

## Slide 2
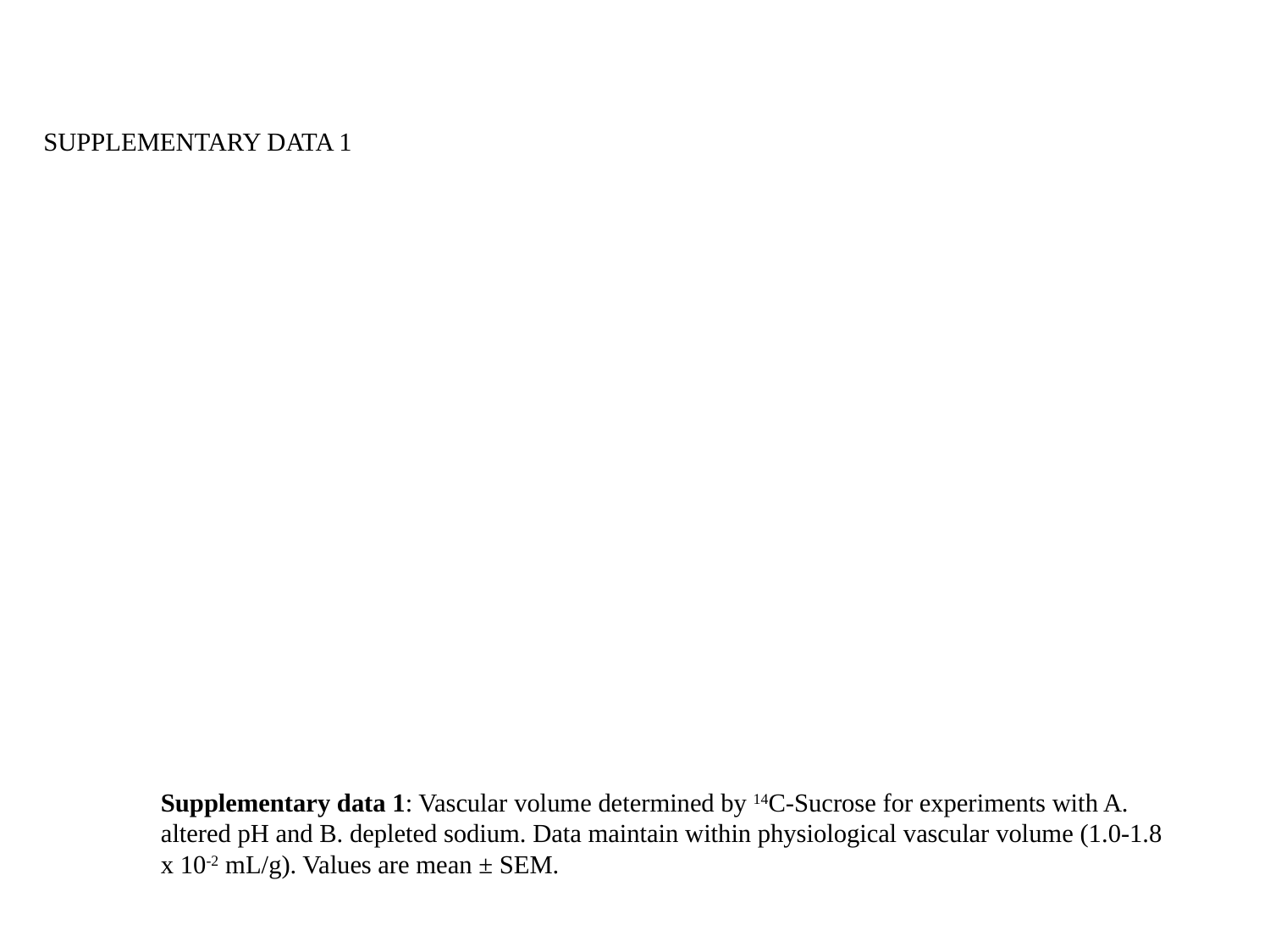

SUPPLEMENTARY DATA 1
Supplementary data 1: Vascular volume determined by 14C-Sucrose for experiments with A. altered pH and B. depleted sodium. Data maintain within physiological vascular volume (1.0-1.8 x 10-2 mL/g). Values are mean ± SEM.

## Slide 3
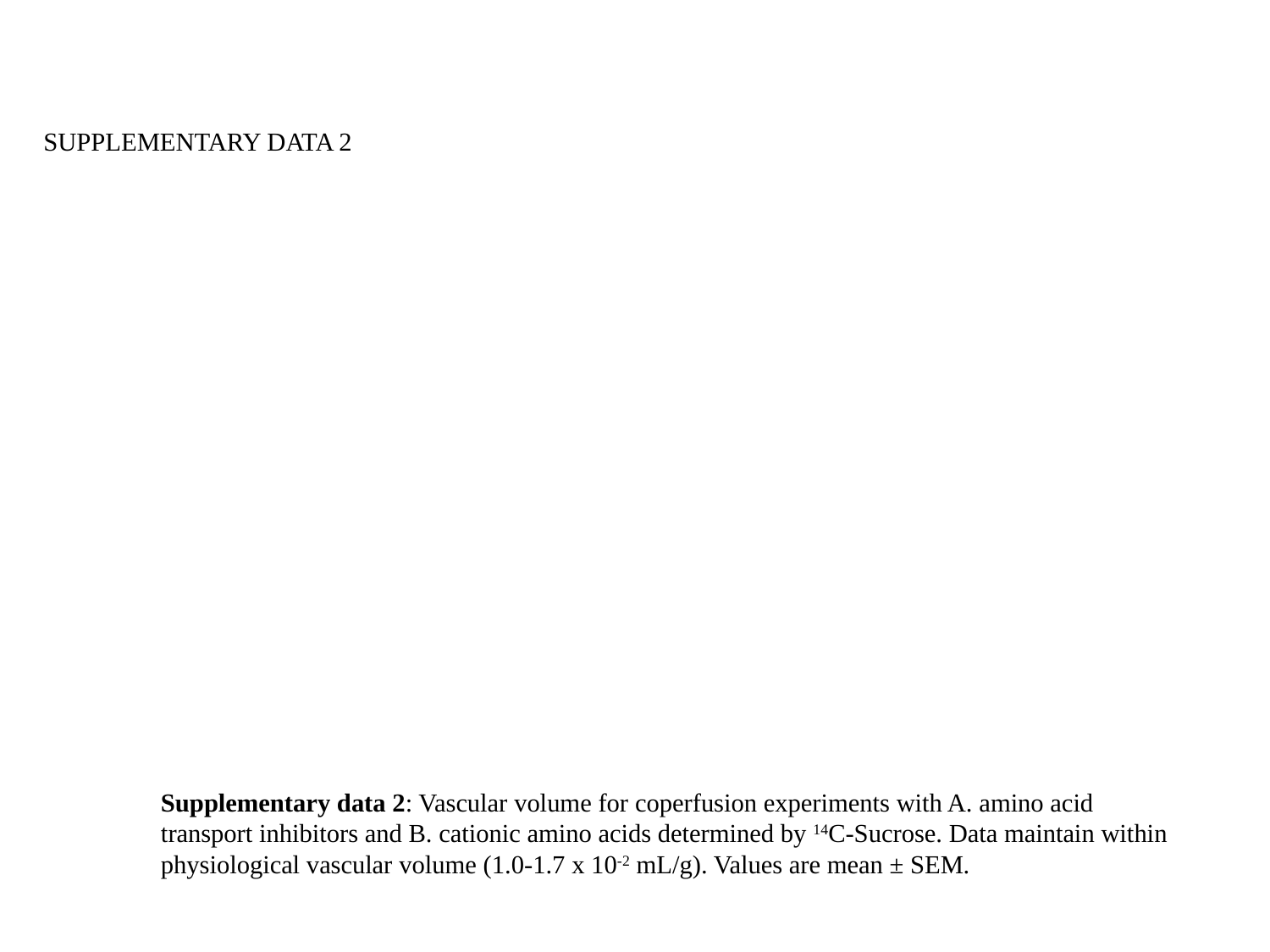

SUPPLEMENTARY DATA 2
Supplementary data 2: Vascular volume for coperfusion experiments with A. amino acid transport inhibitors and B. cationic amino acids determined by 14C-Sucrose. Data maintain within physiological vascular volume (1.0-1.7 x 10-2 mL/g). Values are mean ± SEM.
